# Antibiotic use prior to attending a large diarrheal disease hospital among preschool children suffering from bloody or non-bloody diarrhea: A cross-sectional study conducted in Bangladesh

**DOI:** 10.1371/journal.pone.0314325

**Published:** 2024-11-26

**Authors:** Syed Jayedul Bashar, Md. Ridwan Islam, Sharika Nuzhat, Rukaeya Amin, Md. Mushfiqur Rahman, Patricia B. Pavlinac, Samuel L. M. Arnold, Amy Newlands, Tahmeed Ahmed, Mohammod Jobayer Chisti

**Affiliations:** 1 Nutrition Research Division, International Centre for Diarrheal Disease Research, Bangladesh (icddr,b), Mohakhali, Dhaka, Bangladesh; 2 Clinical and Diagnostic Services, International Centre for Diarrheal Disease Research, Bangladesh (icddr,b), Mohakhali, Dhaka, Bangladesh; 3 Department of Global Health, University of Washington, Seattle, United States of America; 4 Department of Pharmaceutics, University of Washington, Seattle, United States of America; 5 GSK, London, United Kingdom; 6 Office of the Executive Director, International Centre for Diarrheal Disease Research, Bangladesh (icddr,b), Mohakhali, Dhaka, Bangladesh; Suez Canal University, EGYPT

## Abstract

**Background:**

Among diarrheal children, injudicious use of antibiotics is a major public health concern particularly in low- and middle-income countries. There are evidence-based guidelines by the World Health Organization (WHO) to prescribe antibiotics for bloody diarrhea in children. There is a scarcity of published data regarding the judicious use of antibiotics for bloody diarrhea in children. So, we aimed to evaluate the presenting features of bloody diarrhea at hospital with prior antibiotic use at home and the prevalence of injudicious antibiotic use for bloody diarrhea in children.

**Methods:**

We screened 7,289 children aged 24–59 months with diarrhea (≥3 loose stools in the last 24 h) at Dhaka Hospital, International Centre for Diarrheal Disease Research, Bangladesh (icddr,b), from December 5, 2021 to February 16, 2023. Antibiotic intake at home due to current diarrheal illness was evaluated and confirmed by direct observation of a prescription, the bottle of antibiotics, or asking the caregiver about the name of antibiotics.

**Results:**

Out of 7,289 children presented with diarrhea, 3,823 (52.45%) children consumed antibiotics before visiting hospital. 254 (3.48%) children presented with bloody diarrhea, among which 162 ingested antibiotics. Among 162 children, 88 (54.32%) received inappropriate antibiotics due to bloody diarrhea, according to the WHO guidelines. The most prevalent single antibiotic consumed in bloody diarrhea was metronidazole (n = 45, 27.78%), followed by ciprofloxacin (n = 39, 24.07%) and azithromycin (n = 32, 19.75%). After adjusting for relevant covariates like age, sex, presence of straining/tenesmus, fever during admission, history of cough, stunting, wasting, and underweight; children suffering from bloody diarrhea had 1.55 times higher odds of using metronidazole alone or in combination with other antibiotics (aOR:1.55, 95% CI: 1.10–2.19, p-value = 0.012) and 1.93 times higher odds of using multiple antibiotics (aOR:1.93, 95% CI: 1.23–3.02, p-value = 0.004) compared to children with non-bloody diarrhea.

**Conclusion:**

The study underscores the excessive use of antimicrobials among children with diarrheal illnesses. It is also evident that metronidazole use and multiple antibiotic use are increasing among children due to bloody diarrhea, which is alarming and calls for antibiotic stewardship by regulating bodies in the country.

## Introduction

Diarrhea is the third-leading cause of death in under-five children and accounts for 1.7 billion cases of childhood diarrheal illness, with 443, 832 deaths annually reported in 2024 by the World Health Organization (WHO) [1]. Any diarrheal episode in which visible blood is present in the loose or watery feces is diagnosed clinically as bloody diarrhea, or dysentery [1]. Bloody diarrhea is the hallmark of enteric infections mostly caused by invasive bacteria that could spread through the infected person’s excreta via direct fecal-oral contamination [2]. In Bangladesh from 2000 to 2012, acute bloody diarrhea accounted for 27.8% of all diarrheal death among children aged 1 to 4 years [3]. World Health Organization (WHO)’ -recommended management for diarrheal disease includes fluid and electrolyte replacement therapy along with zinc rather than using antimicrobials [4]. WHO makes two exceptions for antibiotic use, for suspected *Shigella* (suspected in cases of dysentery) to prevent its ramifications including life-threatening neurological sequelae [5, 6], and for suspected cholera to reduce the duration of diarrhea. Appropriate detection and monitoring of resistance trends are essential for determining the effective antibiotic for shigellosis treatment [7]. Due to the combined effects of a low socio-economic setting, financial conditions, and paucity of diagnostic resources, it is challenging to find out the causative organisms for diarrhea and an even taller order to determine antibiotic susceptibility [8]. As a result, unjustified (antibiotics used when they shouldn’t be and/or wrong antibiotics) antimicrobials are prescribed empirically [9]. The WHO’s recommended first-line treatment for all cases of bloody diarrhea, including suspected Shigellosis, is ciprofloxacin, and recommended second-line antibiotics are pivmecillinam, azithromycin, and ceftriaxone [10]. In Bangladesh, around 70% of *Shigella* isolates are resistant to ciprofloxacin [11], while 2–5% were found to be resistant to ceftriaxone in an earlier study [12]. Though some authorities recommended azithromycin as the first line treatment for Shigellosis [13], the 2020 report from the surveillance system of a large diarrheal disease hospital in Bangladesh shows that around 50% of *Shigella* species are resistant to azithromycin, which is significantly higher compared to the previous year [11]. All these antibiotics are used for different enteric infections, including shigellosis and enteric fever, and the gradual increase in resistance to these antibiotics throws a burning question at the public health sector [11]. The use of irrational antibiotics is quite high in low- and middle-income countries like Bangladesh [14, 15]. Data is limited regarding the prevalence of antibiotic use prior to attending the hospital for bloody diarrhea among Bangladeshi children aged 24–59 months. So, we aimed to investigate the percentage of antibiotic use among children who are suffering from bloody or non-bloody diarrhea. Along with this, we also evaluated the independent association between bloody diarrhea and subsequent use of different antibiotics.

## Methods

### Study design

**Fig 1:**
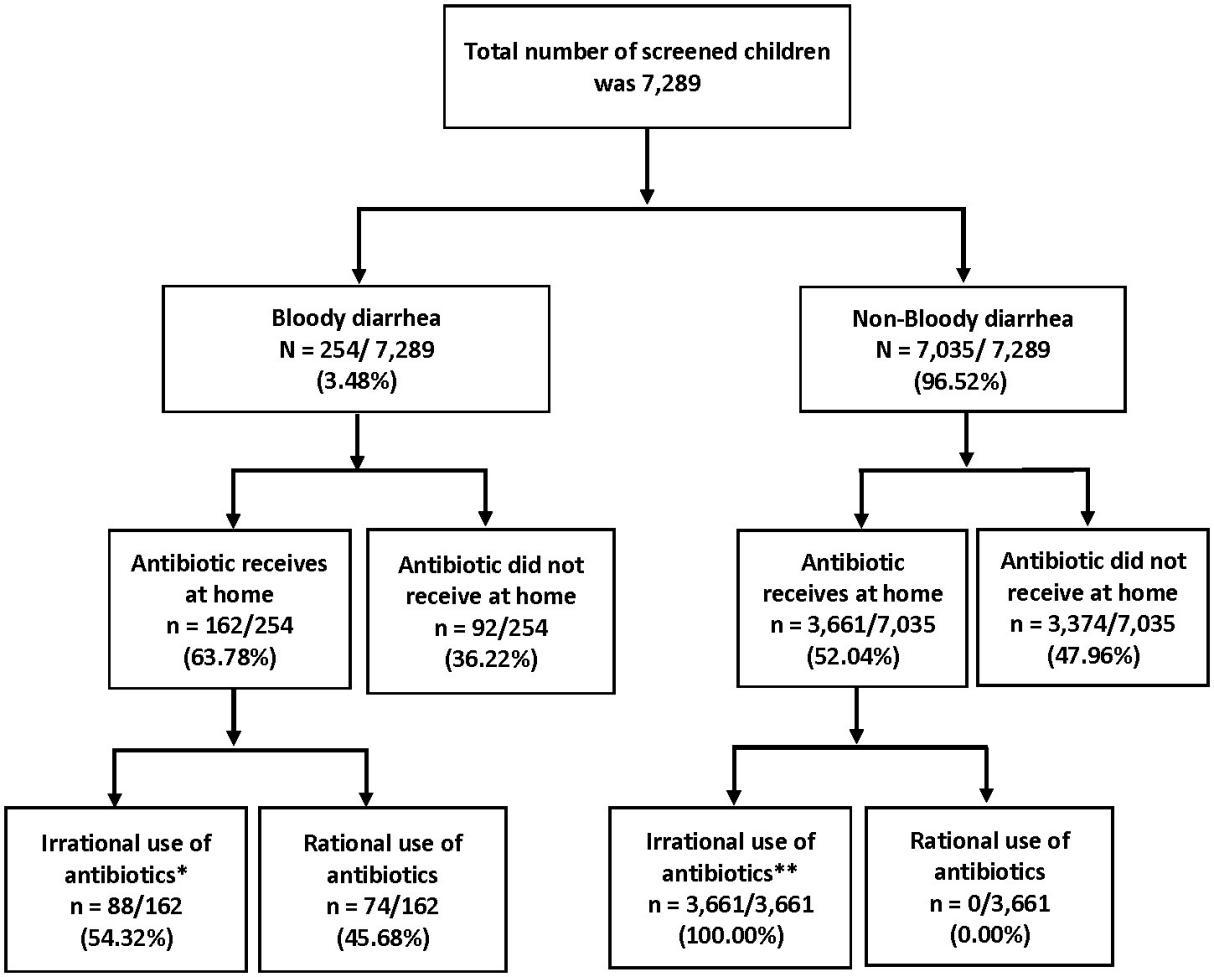
Sampling frame of the selected participants in the analysis. *: Use of antibiotics other than ciprofloxacin/ azithromycin/ ceftriaxone/ pivmecillinam **: Use of any antibiotic.

This cross-sectional study took place at Dhaka Hospital, International Centre for Diarrheal Disease Research, Bangladesh (icddr,b). We extracted data from the screening dataset of a clinical trial entitled ‘Tebipenem Trial in Children with Shigellosis’ (clinicaltrials.gov registration: NCT05121974) from December 5, 2021, to February 16, 2023. Total 7,289 children aged 24–59 months were screened, who were brought to Dhaka Hospital for the management of diarrheal illnesses. The sampling frame is presented in the Fig 1. Children who were aged <24 months, >59 months, and whose parents didn’t give consent were excluded. The original clinical trial did not have any relevant inclusion or exclusion criteria that could have affected the interpretation of these data. Detailed inclusion and exclusion criteria of the parent trial can be found here at https://clinicaltrials.gov/study/NCT05121974.

As soon as the patients arrived at the hospital, doctors and trained healthcare professionals assessed their morbidity and nutritional state. After obtaining informed consent, a licensed physician with the help of trained health-care workers conducted a clinical evaluation of different clinical indicators (severe acute malnutrition, congenital anomaly, systemic illness, septic shock) at the time of screening. In order to confirm whether the child consumed antimicrobial agents for the current diarrheal illness, the caregivers were asked specific questions according to a previous study conducted at Dhaka Hospital, icddr,b [8]. If the caregiver could inform us the specific name of the drug correctly, present the medicine bottle with the name of the antibiotic displayed on it, or show the prescription from a registered physician, we considered it to be accurate. Along with this, if the caregiver failed to apprise the mentioned techniques but could describe the correct method of reconstitution by which he prepared the medicine and tell the price of the medicine that matched with the market price of an antibiotic, we considered the case to have consumed antibiotic, but these cases fell under ‘can’t name’ category.

### Operational definitions

#### Diarrhea

Three or more loose or liquid stools in the last 24 hours [16].

#### Bloody diarrhea

History of presence of visible blood in the loose or watery feces during current diarrheal illness as reported by the caregiver [17].

#### Nutritional status

It was considered according to the WHO Child Growth Standards median for the same age and sex [18].

#### Stunting

Height for age z-score (HAZ) ≤ -2 Standard Deviation (SD).

#### Severe stunting

Height for age z-score (HAZ) < -3 SD.

#### Underweight

Weight for age z-score (WAZ) ≤ -2 SD.

#### Severe underweight

Weight for age z-score (WAZ) < -3 SD.

#### Wasting

Weight for height z-score (WHZ) ≤ -2 SD

#### Severe wasting

Weight for height z-score (WHZ) < -3 SD.

#### Antibiotic used as per guideline

Patients who received ciprofloxacin or azithromycin, or ceftriaxone or pivmecillinam at a time according to the WHO guideline during bloody diarrheal illness [10].

#### Irrational use of antibiotics

- For bloody diarrhea, patients who received antibiotics other than ciprofloxacin or azithromycin or ceftriaxone or pivmecillinam for their current diarrheal illness.
- For non-bloody diarrhea, patients who received any antibiotic for their current diarrheal illness.

#### Use of multiple antibiotics

The current study considered using more than one antibiotic at a time during current diarrheal illness.

### Statistical analysis

STATA for Windows (version 15; StataCorp LLC., Texas, USA) was used to analyze the data. The categorical variables were presented as frequency and percentage and compared using the chi-square (χ^2^) test or Fisher’s exact test. The continuous variables were presented as median (interquartile range, IQR) and compared using the Mann-Whitney U test. Statistical significance was defined as a probability of less than 0.05. By calculating the odds ratios (ORs) and their 95% confidence intervals (CIs), the strength of the association was ascertained. To examine the prevalence of different antibiotics used in bloody and non-bloody diarrhea among children, proportion test (or Binomial proportion test) was performed to assess the use of antibiotics in bloody diarrhea. We conducted multivariable logistic regression model analysis while adjusting for age, sex, presence of straining/tenesmus, fever during admission, history of cough, stunting (HAZ ≤-2), wasting (WHZ ≤-2), and underweight (WAZ ≤-2) to determine the independent association of the exposure variable ‘bloody diarrhea (Yes/No)’ with the dependent variable ‘consumption of the following antibiotics (Yes/No)’: ‘only ciprofloxacin’, ‘only azithromycin’, ‘metronidazole alone or in combination with other antibiotics’, and ‘multiple antibiotics’ prior to presenting to the icddr,b hospital.

#### Ethical consideration

The parent clinical trial was registered under ClinicalTrials.gov (NCT05121974) and the protocol was approved by the Research Review Committee (RRC) and Ethical Review Committee (ERC) which comprises the Institutional Review Board (IRB) of the icddr,b (internal PR-21005). The consent process was approved by the IRB. For our study, we used de-identified data from screening dataset of this clinical trial.

## Results

The study involved 7,289 children aged 24–59 months with loose or watery stool where 254 (3.48%) cases were bloody diarrhea. There were 52.45% (3,823/7,289) of patients who received antibiotics before visiting the hospital. Consumption of antibiotic was found in 63.78% (162/254) of children with bloody diarrhea and in 52.04% (3,661/7,035) of children with non-bloody diarrhea. Among children who consumed antibiotics at home, 54.32% (88/162) and 100% (3,661/3,661) reported irrational antibiotic use for bloody and non-bloody diarrhea respectively (Fig 1). Presenting features of the children who have received antibiotics prior to the hospital visit (n = 3,823) were shown in Table 1. The median (IQR) age of the participants was 32 [27–41] months. Children with bloody diarrhea [36, 29–42] had significantly higher median (IQR) age (p-value = 0.003) than children with non-bloody diarrhea [33, 27–41]. Among the participants, 60.89% were male and most (68.53%) were residents of Dhaka city (Table 1). Overall, 2.46% participants had straining or tenesmus, 35.16% had fever on admission and 3.79% had epilepsy or other convulsive disorders. Straining or tenesmus (29.01% vs 1.28%; p-value<0.001), fever on admission (52.47% vs 34.39%; p-value<0.001), epilepsy or other convulsive disorders (8.64% vs 3.58%; p-value = 0.001) were significantly higher in children with bloody diarrhea than non-bloody diarrheal (Table 1). Among the participants, overall, 9.42% were stunted, 16.48% were wasted and 31.05% were underweight whereas, 2.33% were severely stunted, 4.78% were severely wasted and 8.29% were severely underweight. Stunting was significantly higher (p-value = 0.003) in children with bloody diarrhea (16.05%) than children with non-bloody diarrhea (9.12%). Similarly, wasting was significantly higher (p-value<0.001) in children with bloody diarrhea (29.63%) compared to children with non-bloody diarrhea (15.90%) (Table 1).

**Table 1:** Presenting features of bloody and non-bloody diarrhea among children who have received antibiotics (n = 3823) prior to the hospital visit.

Variables	Overall (n = 3823)	Presenting Features		P value
		Bloody (n = 162)	Non-Bloody (n = 3661)	
**Age in months, median (IQR)**	32 (27–41)	36 (29–42)	33 (27–41)	**0.003**
**Sex (Male), n (%)**	2328 (60.89)	97 (59.88)	2231 (60.94)	0.786
**Residency (inside Dhaka), n (%)**	2620 (68.53)	100 (61.73)	2520 (68.83)	0.057
**Straining or tenesmus (Yes), n (%)**	94 (2.46)	47 (29.01)	47 (1.28)	**<0.001**
**Fever on admission (Yes), n (%)**	1344 (35.16)	85 (52.47)	1259 (34.39)	**<0.001**
**H/O cough (Yes), n (%)**	637 (16.16)	29 (17.90)	608 (16.61)	0.665
**Congenital anomaly disorder (Yes), n (%)**	49 (1.28)	2 (1.23)	47 (1.2)	0.957
Epilepsy or other convulsive disorders* (Yes), n (%)	145 (3.79)	14 (8.64)	131 (3.58)	**0.001**
**Stunting (LAZ <-2) (Yes), n (%)**	360 (9.42)	26 (16.05)	334 (9.12)	**0.003**
**Severe Stunting (LAZ <-3) (Yes), n (%)**	89 (2.33)	5 (3.09)	84 (2.29)	0.513
**Underweight (WAZ <-2) (Yes), n (%)**	1187 (31.05)	59 (36.42)	1128 (32.42)	0.125
**Severe Underweight (WAZ <-3) (Yes), n (%)**	317 (8.29)	17 (10.49)	300 (8.19)	0.319
**Wasting (WLZ <-2) (Yes), n (%)**	630 (16.48)	48 (29.63)	582 (15.90)	**<0.001**
**Severe Wasting (WLZ <-3) (Yes), n (%)**	183 (4.78)	11 (6.79)	172 (4.70)	0.230

**Fig 2:**
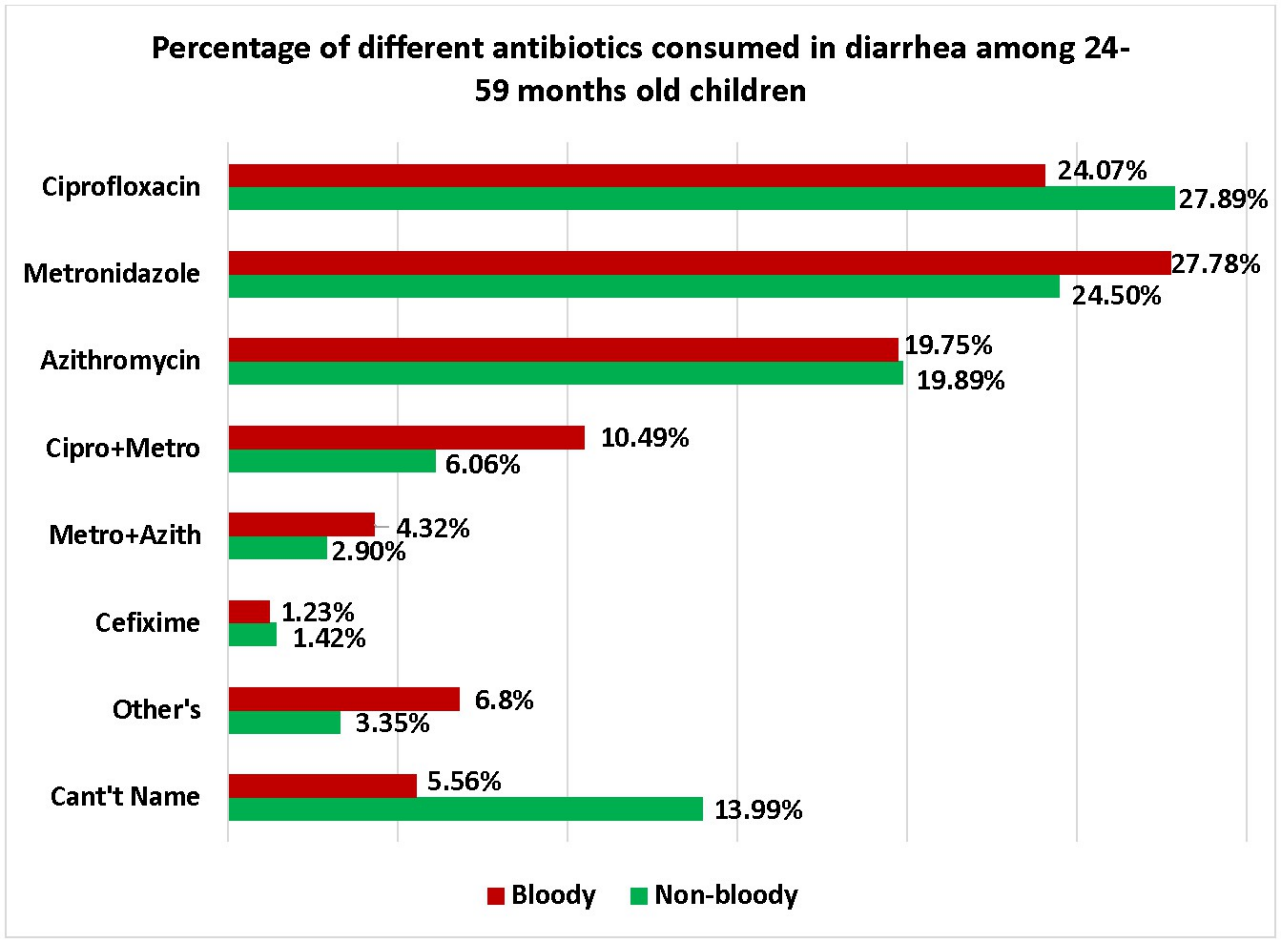
Spectrum of antibiotics consumed in diarrheal children.

The highest used single antibiotic in bloody diarrhea was metronidazole (n = 45, 27.78%), followed by ciprofloxacin (n = 39, 24.07%), azithromycin (n = 32, 19.75%), and cefixime (n = 2, 1.23%). The predominant antibiotic combination was ciprofloxacin + metronidazole (n = 17, 10.5%), subsequently metronidazole + azithromycin (n = 7, 4.32%) and then ciprofloxacin + metronidazole + azithromycin (n = 3, 1.85%) (Fig 2).

Here, we included the "Other" antibiotics, encompassing ceftriaxone, erythromycin, ciprofloxacin + erythromycin, and ciprofloxacin + nitazoxanide, collectively accounting for 6.80% of all antibiotics administered. However, in non-bloody diarrhea, ciprofloxacin (n = 1,021, 27.89%) was the most commonly used antibiotic later on metronidazole (n = 897, 24.50%), azithromycin (n = 728, 19.89%), and cefixime (n = 52, 1.42%). The predominant antibiotic combination was ciprofloxacin + metronidazole (n = 222, 6.06%), then metronidazole + azithromycin (n = 106, 2.90%) among non-bloody diarrheal children (Fig 2). Here, the category of "Other" antibiotics included ceftriaxone, erythromycin, sulfamethoxazole-trimethoprim, ciprofloxacin + azithromycin, ciprofloxacin + erythromycin, ciprofloxacin + cefixime, ciprofloxacin + nitazoxanide, metronidazole + erythromycin, metronidazole + cefixime, metronidazole + nitazoxanide, metronidazole + ceftriaxone, azithromycin + erythromycin, and azithromycin + nitazoxanide, collectively representing 3.35% of all antibiotics administered. Overall, 13.62% (521/3,823) of the caregivers of children who consumed antibiotics were unable to name the antibiotic thus categorized as “can’t name” (Fig 2) whereas in bloody diarrhea it was 5.55% (9/162) and in non-bloody diarrhea it was 13.98% (512/3,661).

Considering the prevalence of various antibiotics used, consumption of metronidazole alone or in combination with other antibiotics was significantly higher (p-value = 0.005) in bloody diarrheal children (44.44%, 95% CI: 36.71–52.44) compared to children with non-bloody diarrhea (33.46%, 95% CI: 31.94–35.02). Multiple antibiotic consumption, regardless of the type was, also significantly higher (p-value<0.001) in bloody diarrheal children (19.14%, 95% CI: 13.56–26.22) than non-bloody diarrheal children (10.49%, 95% CI: 9.52–11.54). However, the individual uses of ciprofloxacin, azithromycin, and ceftriaxone were found greater in non-bloody diarrheal children than bloody diarrheal children, but the association was not significant (Table 2).

**Table 2:** Prevalence of different antibiotics used in bloody and non-bloody diarrhea among children (n = 3,823) aged 24–59 months.

Antibiotic	Overall (n = 3823)		Bloody (162)		Non-Bloody (3,661)		p-value
	Frequency, N	Percentage, % (95% CI)	Frequency, N	Percentage, % (95% CI)	Frequency, N	Percentage, % (95% CI)	
Only Ciprofloxacin*	1060	27.73 (26.31–29.18)	39	24.07 (17.87–31.54)	1021	27.89 (26.45–29.38)	0.331
Only Azithromycin*	760	19.88 (18.62–21.18)	32	19.75 (14.09–26.89)	728	19.89 (18.61–24.22)	0.999
Only Ceftriaxone**	14	0.37 (0.20–0.61)	3	1.85 (0.48–5.74)	11	0.30 (0.16–0.55)	0.050
Metronidazole* (alone or in combination with other antibiotics)	1297	33.93 (32.43–35.45)	72	44.44 (36.71–52.44)	1225	33.46 (31.94–35.02)	**0.005**
Use of multiple antibiotics***	415	10.86 (9.89–11.88)	31	19.14 (13.56–26.22)	384	10.49 (9.52–11.54)	**<0.001**

* Proportion test;

** Binomial proportion test;

***Use of multiple antibiotics: The current study considered using more than one antibiotic as “Yes”, otherwise “No”

After adjusting for age, sex, presence of straining/tenesmus, fever during admission, history of cough, stunting (HAZ ≤-2 SD), wasting (WHZ ≤-2 SD), and underweight (WAZ ≤-2 SD); children with bloody diarrhea had 55% higher odds of using metronidazole (adjusted Odds Ratio, aOR:1.55, 95% CI: 1.10–2.19, p- value = 0.012) than those with non-bloody diarrhea. Moreover, children with bloody diarrhea had 93% higher odds of using multiple antibiotics (aOR:1.93, 95% CI: 1.23–3.02, p- value = 0.004) compared to children with non-bloody diarrhea. However, use of ciprofloxacin (aOR:0.89, 95% CI: 0.60–1.32, P-value = 0.560) and azithromycin (aOR:0.94, 95% CI: 0.61–1.43, p-value = 0.763) had no significant association with bloody diarrhea (Table 3).

**Table 3:** Multivariable logistic regression models to evaluate the independent association between incidence of bloody diarrhea and the subsequent use of different antibiotics.

Logistic Regression Models	Outcome variables (Antibiotic use)	Unadjusted OR		Adjusted OR*	
		OR (95% CI)	p-value	aOR (95% CI)	p-value
1	Only Ciprofloxacin	0.82 (0.57–1.18)	0.289	0.89 (0.60–1.32)	0.56
2	Only Azithromycin	0.99 (0.67–1.47)	0.967	0.94 (0.61–1.43)	0.763
3	Metronidazole (alone or in combination with other antibiotics)	1.59 (1.16–2.19)	**0.004**	1.55 (1.10–2.19)	**0.012**
4	Use of multiple antibiotic**	2.02 (1.35–3.03)	**<0.001**	1.93 (1.23–3.02)	**0.004**

OR = odds ratio; aOR = adjusted odds ratio; CI = confidence interval;

*adjusted for age, sex, presence of straining/tenesmus, fever during admission, history of cough, stunting (HAZ ≤-2), wasting (WHZ ≤-2), underweight (WAZ ≤-2)

**Use of multiple antibiotics: The current study considered using more than one antibiotic (any) as “Yes”, otherwise “No”

## Discussion

The current study aimed to investigate the prevalence of different antibiotic use among 24- to 59-month-old children who got admitted to the hospital with diarrheal illnesses. We have also identified which antibiotics are most likely to be used in cases of bloody diarrhea. The WHO guidelines for the Integrated Management of Childhood Illness suggest empirical use of antibiotics only for bloody diarrhea [19] and the use of antibiotics in treating shigellosis has potential to substantially reduce the duration of disease [20]. For the treatment of watery diarrhea in children, oral rehydration and zinc supplementation are advised since viral infections are typically the cause [21]. Moreover, molecular techniques have suggested that a significant portion of instances of watery diarrhea are also caused by bacteria and may benefit from antibiotic treatment [22, 23]. Although, shigellosis is endemic in most developing countries, it is the leading cause of bloody diarrhea all over the world [24] and ciprofloxacin, azithromycin, and ceftriaxone are recommended for this particular form of diarrhea, where the first two are suggested as first-line treatments [11] and the latter, ceftriaxone, is reserved as a second-line treatment option for treatment failure cases [17, 25].

Our study reports that more than 50% of children received antibiotics for non-bloody diarrhea, which is coherent with findings from other studies conducted in Bangladesh, India, and Thailand [8, 26–28]. Considering the WHO guidelines for the use of antimicrobials in bloody diarrhea, we found that 54.32% of patients in our study consumed antibiotics that were not recommended by the WHO, most of which consisted of metronidazole, cefixime, erythromycin, amoxicillin, cefuroxime, flucloxacillin, etc. In a low-and middle-income (LMIC) country like Bangladesh, the increased use of irrational antibiotics is alarming. It is understandable that there are multiple factors working towards this striking issue. In a LMIC like our country, drugs are available to patients from different sources; for example, local pharmacies, licensed hospital drug stores, drug distribution centers, roadside medicine shops, and traditional healers [9, 29, 30]. Although getting access to antibiotics without a prescription is considered illegal, this is actually not difficult but rather a common scenario [8]. An old but important review article found that lack of knowledge regarding antibiotic prescribing among healthcare practitioners, mistrust in or delayed laboratory investigation results, doctors’ desire to be on the safe side in order to meet patients’ demands, and social beliefs on antibiotic use are the key determining factors that influence antimicrobial prescribing practice [31]. In a study conducted in Peru, it was reported that although physicians had adequate knowledge of diarrheal management, they advised antibiotics to most of the patients. Even though the patients confirmed they were not passing bloody stool, physicians prescribed antimicrobials in absence of confirmatory investigations [32]. Empirical drug prescription is also quite common in LMICs like Bangladesh, where a diagnostic approach is considered irrelevant and costly by the populace. Laboratory investigations are performed only when the initial medicine fails to treat the patient [33]. People also prefer to purchase medicines directly from the pharmacy without prior consultation with a doctor as theses drug shops provide immediate treatment as well as easy access to medicines [34]. Lack of knowledge and awareness of drug sellers, acting on customer demand, and improper practice of law are also some of the key determinants that increase irrational antibiotic use [9]. Such inappropriate use of antibiotics can cause antimicrobial resistance, damage to beneficial gut flora, prolonged illness, extended pathogen status, and an increased risk of infection [35].

One of the interesting findings of our study was that consumption of metronidazole was significantly higher among children with bloody diarrhea. Metronidazole is the choice of drug in suspected amebiasis along with giardiasis cases that produce non-bloody diarrhea [17, 36], thus the use of metronidazole in bloody diarrhea is not recommended by the WHO as most bloody diarrhea occurs due to shigellosis. So, a targeted approach in the case of antibiotic selection is suggested. Metronidazole is a drug that is commonly used for the treatment of diarrhea in Bangladesh and is generally well known among people [8]. This drug is cheaper than WHO recommended antibiotics and easily available at local pharmacies, where drug sellers present metronidazole as a potential antibiotic choice for patients. A Bangladeshi study found that metronidazole prescribing rates were equally high among registered physicians and non-registered healthcare assistants [37]. We can also hypothesize that, as metronidazole was used for the treatment of dysentery previously as sanitation and hygiene was sub-standard in old days, the doctors that are not updated with the guidelines in the era of improved sanitation, still prescribe metronidazole to their patients. Also, patients from lower socio-economic status may prefer metronidazole over ciprofloxacin and azithromycin, as these are priced at a higher range compared to metronidazole.

Though the use of antimicrobials is found to be effective in cases of bacterial diarrhea, antibiotic use particularly in non-infectious or viral diarrhea, can lead to unfavorable clinical outcomes [38, 39]. The primary mechanisms involved, in such cases, were overgrowth of pathogenic bacteria like *Clostridium*, *Klebsiella*, and *Candida*, and impaired immune responses and metabolic alterations [40–42]. Early-life antibiotic use has been attributed to gut microbiota dysbiosis, which may increase susceptibility to malnutrition and various acute and chronic illnesses [43–45]. Disrupting the delicate balance of the gut microbiota can affect crucial functions, including metabolism, immune modulation, nutritional development, energy regulation, and intestinal mucosal integrity, all of which are essential for overall health [44, 46]. Dysbiosis in the gut microbiota due to antibiotics has been associated with both intestinal and systemic diseases, with inappropriate or excessive use leading to a higher risk of systemic fungal infections [46]. Broad-spectrum antibiotic use, especially in extreme age groups, poses an even greater risk for systemic fungal infections like, candidiasis [47]. Pediatric intensive care patients, particularly in intensive care settings, are especially vulnerable due to their fragile immune systems and frequent exposure to antimicrobials [48]. Our study also found that the use of multiple antibiotics is significantly more common among patients who are suffering from bloody diarrhea. The ineffectiveness of metronidazole in treating the illness, coupled with the diminishing efficacy of ciprofloxacin and azithromycin against *Shigella* species, contributes significantly to the challenge of addressing the condition [11]. So, patients are switching antibiotics randomly, hoping one of them might work before finally visiting the hospital.

Our study has a few limitations. Due to the cross-sectional nature of the study, a causal relationship could not be established. So, findings from this study should be generalized cautiously when considered for the overall population. The class distribution for outcome variable was skewed and weighting was not done. The bloody diarrhea arm had smaller sample compared to the non-bloody arm. The Ceftriaxone model had fewer cases so was not included in the final analysis. Bloody diarrhea was considered based on the clinical features. No culture data were available, and thus, causative organisms could not be identified. Data on children aged less than two years were not available; otherwise, it would have been possible to present a complete picture of under-five diarrheal patients. Along with these factors, data on socioeconomic status, WASH (water, sanitation, and hygiene) practices, parent’s education, child’s birth order, dehydration status, vaccination profile and breastfeeding history were also unavailable.

## Conclusion

In conclusion, our study draws attention to the prevalence of irrational antibiotics used among 24- to 59-month-old children suffering from bloody diarrhea as well as non-bloody diarrhea in Bangladesh. The unwarranted use of metronidazole in bloody diarrhea may contribute to the emerging multi-drug resistance of invasive diarrheal pathogens. Despite the fact that one-third of bloody diarrheal cases were treated by following proper guidelines, two-thirds of them are still ignoring the WHO recommendation. In consideration of our findings, we suggest that the Bangladesh government, stakeholders, and researchers intensify their efforts to implement a nationwide antimicrobial stewardship program and to raise public awareness to help prevent the irrational use of antibiotics in bloody diarrhea.

## Supporting information

S1 Table Presenting features of bloody and non-bloody diarrhea among children who have received antibiotics (n = 3823) prior to the hospital visit. (DOCX)
