# Supplementary material for: Antibiotic use prior to attending a large diarrheal disease hospital among preschool children suffering from bloody or non-bloody diarrhea: A cross-sectional study conducted in Bangladesh
Source: PLoS One. 2024 Nov 26;19(11):e0314325. doi: 10.1371/journal.pone.0314325 (PMC11593761; doi:10.1371/journal.pone.0314325)
Supplement: S1 Table — (DOCX) [file pone.0314325.s001.docx]

**Supporting Information**

**S1 Table: Presenting features of bloody and non-bloody diarrhea among children who have received antibiotics (n = 3823) prior to the hospital visit**

| **Variables** | **Overall**  **(n = 3823)** | **Bloody**  **(n = 162)** | **Non-Bloody**  **(n = 3661)** | **P value** |
| --- | --- | --- | --- | --- |
| **Age in months;**  **Median (IQR)** | 32 (27, 41) | 36 (29, 42) | 33 (27, 41) | **0.003** |
| **Sex (Male),**  **n (%)** | 2328 (60.89) | 97 (59.88) | 2231 (60.94) | 0.786 |
| **Residency**  **(inside Dhaka),**  **n (%)** | 2620 (68.53) | 100 (61.73) | 2520 (68.83) | 0.057 |
| **Straining**  **or tenesmus**  **Yes, n (%)** | 94 (2.46) | 47 (29.01) | 47 (1.28) | **<0.001** |
| **Fever on admission**  **Yes, n (%)** | 1344 (35.16) | 85 (52.47) | 1259 (34.39) | **<0.001** |
| **H/O cough**  **Yes, n (%)** | 637 (16.16) | 29 (17.90) | 608 (16.61) | 0.665 |
| **Congenital anomaly disorder**  **Yes, n (%)** | 49 (1.28) | 2 (1.23) | 47 (1.2) | 0.957 |
| **Epilepsy or other convulsive disorders***  **Yes, n (%)** | 145 (3.79) | 14 (8.64) | 131 (3.58) | **0.001** |
| **Stunting (LAZ <-2)**  **Yes, n (%)** | 360 (9.42) | 26 (16.05) | 334 (9.12) | **0.003** |
| **Severe Stunting (LAZ <-3)**  **Yes, n (%)** | 89 (2.33) | 5 (3.09) | 84 (2.29) | 0.513 |
| **Underweight (WAZ <-2)**  **Yes, n (%)** | 1187 (31.05) | 59 (36.42) | 1128 (32.42) | 0.125 |
| **Severe Underweight (WAZ <-3)**  **Yes, n (%)** | 317 (8.29) | 17 (10.49) | 300 (8.19) | 0.319 |
| **Wasting (WLZ <-2)**  **Yes, n (%)** | 630 (16.48) | 48 (29.63) | 582 (15.90) | **<0.001** |
| **Severe Wasting (WLZ <-3)**  **Yes, n (%)** | 183 (4.78) | 11 (6.79) | 172 (4.70) | 0.230 |
